# Squamous cell carcinoma of the nail unit after repeated UV nail lamp exposure. A call for action?^☆^

**DOI:** 10.1016/j.abd.2023.07.020

**Published:** 2024-08-06

**Authors:** Tatiana Ordoñez, Marina Ruf, Valeria Angles, Gabriel Brau, Damián Ferrario, Luis Mazzuoccolo

**Affiliations:** Dermatology Department, Hospital Italiano de Buenos Aires, Buenos Aires, Argentina

*Dear Editor*,

An otherwise healthy 26-year-old female consulted Dermatology Service due to 1 year of changes in the color of her nail, and detachment of the nail plate on the fourth left finger. The patient referred had been continuously exposed to UV nail lamps twice a month for two years. She did not use sunscreen or any other form of protection while using the device. She did not use tanning beds either. She had a negative mycologic test performed and received topical mycological treatments with no response.

On examination, proximal leukonychia, distal yellow-brown chromonychia, and onychomadesis were observed (Fig. 1).

**Figure 1:**
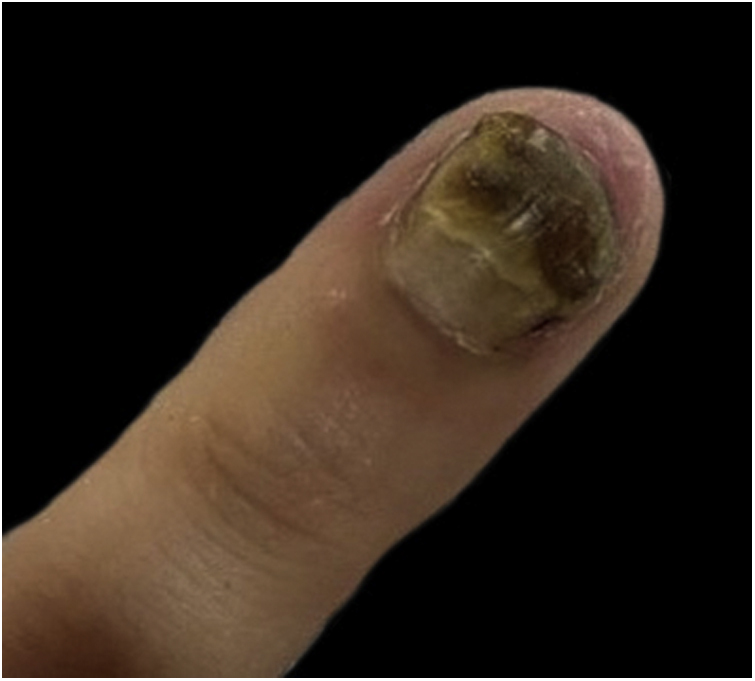
Proximal leukonychia, distal yellow-brown chromonychia, and onychomadesis on the fourth left finger.

A biopsy of the nail matrix was performed, and the histopathology showed hyperparakeratosis, acanthosis, and intraepidermal proliferation of atypical keratinocytes, absence of maturation, and abundant mitoses. A squamous cell carcinoma in situ with partial resection (Fig. 2). Due to the high-risk tumor location, Mohs micrographic surgery was indicated (Fig. 3).

**Figure 2:**
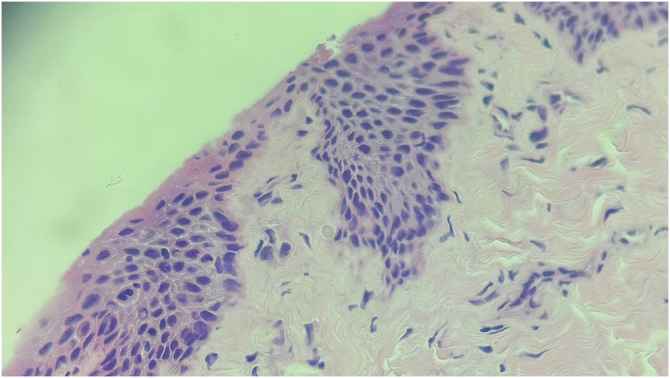
Histopathology. Hyperparakeratosis and intraepidermal proliferation of atypical keratinocytes with abundant mitoses (Hematoxylin & eosin, 40x).

**Figure 3:**
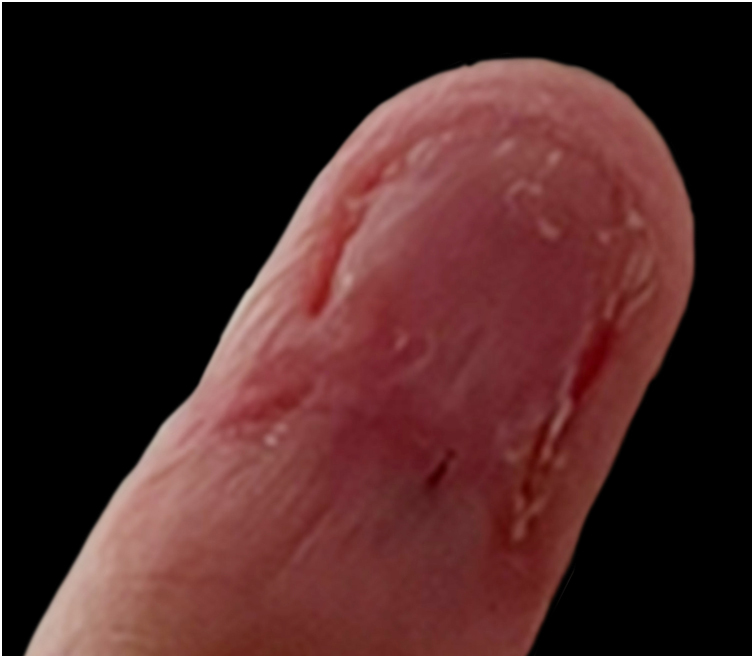
Postoperative result. A scar on the nail bed is observed.

Squamous cell carcinoma is a malignant tumor of keratinizing cells in the epidermis and its appendages. Well-defined risk factors exist for its development, with the main one being exposure to Ultraviolet Radiation (UVR).^1^ Within the UVR spectrum, type A and its association with squamous cell carcinoma is well-known after prolonged exposure.^2^

Currently, gel or acrylic manicure is a very common practice in the population that requires UVA with fluorescent or LED lamps that have an emission spectrum of 375 to 425 nanometers.^3^ Currently, some case reports suggest a link between the use of UVA nail lamps and the development of squamous cell carcinomas and actinic keratosis, either on fingers or hands dorsum.^4^, ^5^, ^6^, ^7^ The literature and case reports published so far conclude that the risks are potential and are limited to giving recommendations on their use. In a recently published study on Nature Communications, the molecular effect of the radiation emitted by UV nail lamps was experimentally evaluated in mammalian and human cells, demonstrating that it is cytotoxic, genotoxic, and mutagenic, predisposing to an increased risk of carcinomas.^2^

Some authorities such as the Food and Drug Administration and the World Health Organization mention the risk of carcinomas due to exposure to ultraviolet radiation, either from the sun or artificial sources such as tanning beds. Still, they do not mention lamps used for manicures.^8^, ^9^

With this case presentation, while we can’t assert a declared relationship between the use of UV lamps and the development of squamous cell carcinoma in our patient, it was highly suggestive due to being a young woman without other risk factors, with the presence of a single presentation tumor in an area directly exposed to nail-curing lamps.

In light of permanent manicures and acrylic nails becoming increasingly popular, we consider it important to analyze the issue due to the potential impact this practice could have, especially on young people unaware of the possible risks of this habit.

## Financial support

None declared.

## Authors’ contributions

Tatiana Ordoñez: The study concept and design; Writing of the manuscript.

Marina Ruf: The study concept and design; Intellectual participation in the propaedeutic and/or therapeutic conduct of the studied cases.

Valeria Angles: Data collection, or analysis and interpretation of data.

Gabriel Brau: Writing of the manuscript or critical review of important intellectual content.

Damián Ferrario: Data collection.

Luis Mazzuoccolo: Final approval of the final version of the manuscript.

## Conflicts of interest

None declared.
